# Supplementary material for: SCONCE2: jointly inferring single cell copy number profiles and tumor evolutionary distances
Source: BMC Bioinformatics. 2022 Aug 19;23:348. doi: 10.1186/s12859-022-04890-w (PMC9392257; doi:10.1186/s12859-022-04890-w)
Supplement: Supplementary file 1 — Additional file 1. This file contains supplementary figures showing the simulated tree structures, analyses from summarizing over the nearest 10 cells, two examples of CNA detection in real data, and Robinson-Foulds distances across more copy number profiles and from summarizing across the nearest 10 cells. Additionally, it contains tables showing simulation parameter details, full median breakpoint distances and omega values (across all cells and across the 10 nearest cells), median pairwise Euclidean distances between SCONCE profiles, and median Robinson-Foulds distances (across all cells and across the 10 nearest cells). [file 12859_2022_4890_MOESM1_ESM.pdf]

Supplementary Notes and Figures

Tree illustrations

We simulated 128 cells from four distinct tree structures, described in Simulations. Small 8 cell trees are shown for illustration purposes in Supplementary Figure S1, Additional File 1. Tree A is ultrametric and maximally imbalanced, tree B is ultrametric and perfectly balanced, tree C is not ultrametric and maximally imbalanced with uniform branch lengths, and tree D is not ultrametric and maximally imbalanced with logarithmically decaying branch lengths.

SSE and breakpoint distance accuracy over the nearest 10 cells

Across parameter sets, the median sum of squared error (SSE) between the true and inferred copy number profile for each cell is lowest when each cell's consensus profile is calculated by summarizing across the nearest 10 cells, rather than over all 20 in each subset, as rare events aren't averaged out. The nearest cells are determined by the lowest Eucliden distance on each cell's SCONCE profile. Similar to Figure 3, SSE is shown across parameter sets for CNPs from SCONCE (independent inference); from each pairwise analysis; summarized across pairs using mean, median, and mode; and from AneuFinder in Supplementary Figure S2, Additional File 1.

Additionally, similar to Figure 4, we show breakpoint detection accuracy in Supplementary Figure S3, Additional File 1 when consensus profiles are calculated by summarizing over each cell's nearest 10 cells. Median breakpoint distance and $\omega = \frac{\#\text{ inferred breakpoints}}{\#\text{ true breakpoints}}$ values are given in Supplementary Tables S5 and S6, Additional File 1.

Median Breakpoint Distance and ω Values

To evaluate breakpoint detection accuracy, we summed the distance from each true breakpoint to its nearest inferred breakpoint. We define $\omega = \frac{\#\text{ inferred breakpoints}}{\#\text{ true breakpoints}}$, such that highest accuracy is indicated by low breakpoint distance and ω close to 1. Median breakpoint distance and ω values are given across all analyzed cells for each tree in Supplementary Tables S2 and S3, Additional File 1. Similarly, median breakpoint distance and ω values when consensus methods only summarize across the 10 nearest cells are given in Supplementary Tables S5 and S6, Additional File 1.

Robinson-Foulds Distance Values

SCONCE2 estimates branch lengths for tree $\mathcal{T} = [t_1, t_2, t_3]$ for every pair of cells. To evaluate the usefulness of $t_2 + t_3$ as a distance metric for phylogeny building, we first calculated distance matrices using $t_2 + t_3$. For comparison, we calculated distance matrices using several different distance metrics (Euclidean distance, cnp2cnp, and MEDICC) at different points in the SCONCE2 pipeline (after SCONCE (independent inference); on summarized CNPs from the mean, median, and mode). As a sanity check, we also calculated distance matrices on the true simulated CNPs (i.e., removing all noise from the copy number inference process). Next, we applied neighbor-joining to these distance matrices and calculated the Robinson-Foulds (RF) distance between each resulting tree and the true simulated tree. For each

cell subset in each tree, RF distances are plotted in Supplementary Figure S6, Additional File 1, and median RF distances are given in Supplementary Table S7, Additional File 1.

Using the Euclidean distance is remarkably stable to variations in CNPs. Both the cnp2cnp and MEDICC metrics had the highest median RF distances for SCONCE, and the lowest on the true CNPs, with distances from mean, median, and mode lying between those two extremes. Across all parameter sets, using $t_2 + t_3$ resulted in median RF distances lower than other methods, with the exceptions of using the true CNPs. However, it stands to reason that one would not have access to noiseless copy number profiles in experimental conditions.

Additionally, we calculated the above distance matrices and ran neighbor-joining on CNPs where consensus profiles were summarized across only the nearest 10 cells. However, analyzing only the nearest 10 cells means some cell pairs get skipped, and therefore do not have an $t_2 + t_3$ estimate. To address this, we only calculated RF distances on trees estimated from other metrics.RF distances are plotted in Figure S7, and median RF distances are given in Supplementary Table S8, Additional File 1.

Detailed SCONCE2 pipeline

The SCONCE2 pipeline, shown in Figure 2, consists of four main steps: 1) Tumor cells are first independently analyzed through SCONCE to estimate model parameters (for cell A, $\{s_A, \alpha_A, \beta_A, \gamma_A, t_A\}$) and cell specific copy number profiles. 2) Shared parameter estimates, $\{\alpha, \beta, \gamma\}$, are then summarized across all cells using the median. 3) The branch lengths in tree $\mathcal{T} = [t_1, t_2, t_3]$ are then independently estimated for each pair of cells. Finally, the Viterbi decoding is used to calculate copy number profiles for each pair of cells, and 4) cell specific copy number profiles are summarized across pairs. Optionally, only the κ nearest neighbor cells (defined by the Euclidean distance on the SCONCE copy number profiles) are summarized for a given cell.

Supplementary Figures

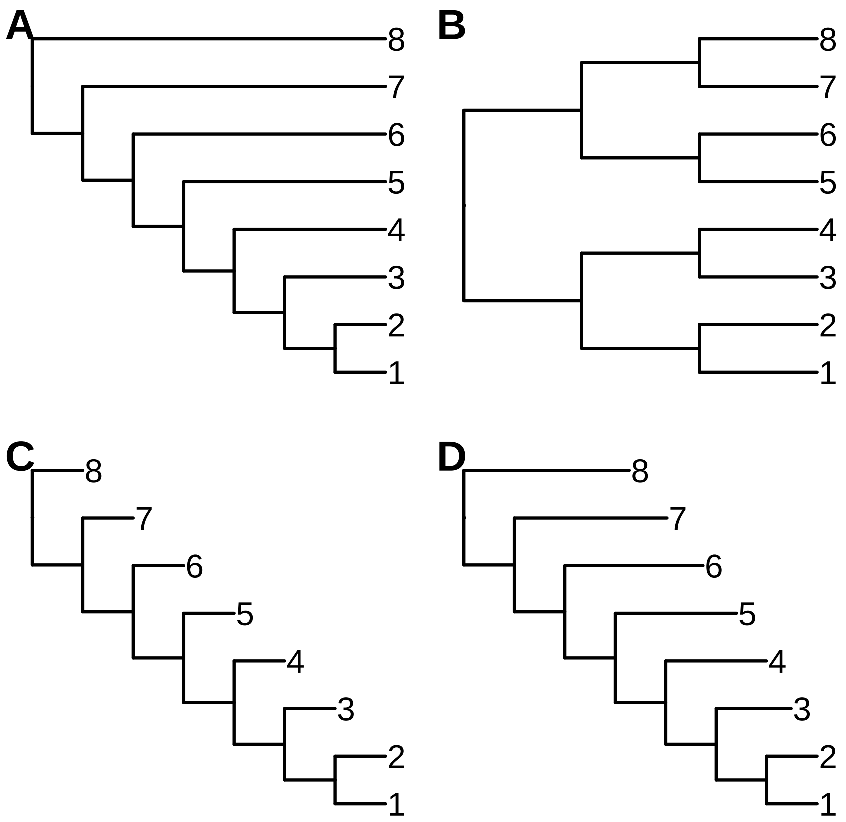

Figure S1: Tree structure for each simulated data set. Although each tree contained 128 cells, only 8 cells for each tree is shown for brevity.

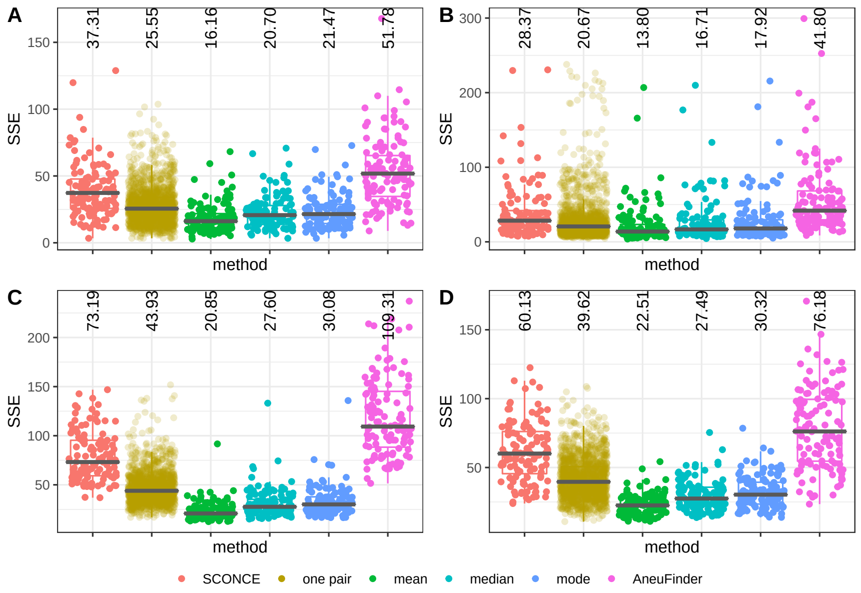

Figure S2: Boxplots of SSE across parameter sets and CNP calling method. Here, for summary methods (mean, median, and mode), each cell's consensus CNP is calculated over its nearest 10 cells. Each dot shows one cell, CNP calling method is on the x-axis, and SSE is on the y-axis, with median SSE values printed at the top of each column. The median SSE is indicated with a black bar and printed at the top of each column. Across parameter sets, calculating consensus calls results in lower SSE than both competing methods and summarizing over all pairs of cells (Figure 3).

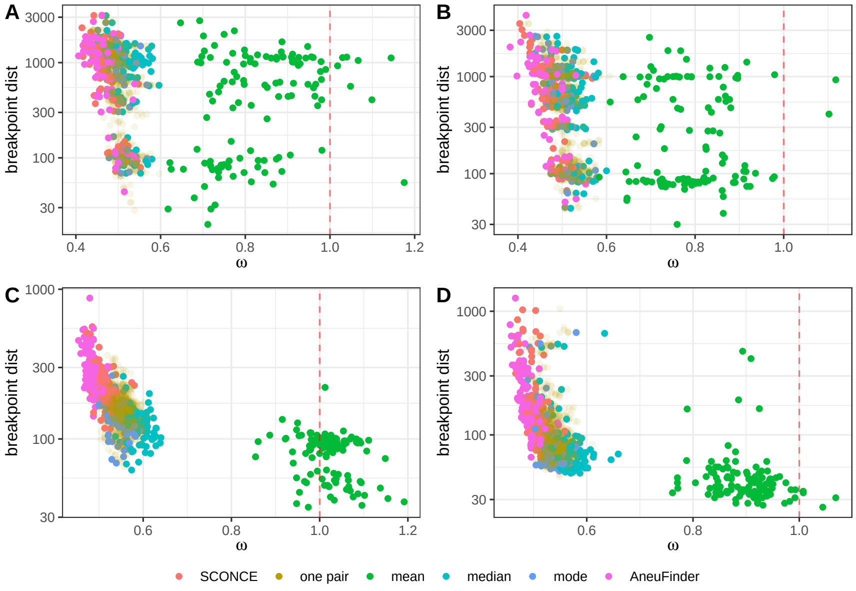

Figure S3: Breakpoint detection accuracy across parameter sets and CNP calling methods, where consensus CNPs are summarized over each cell's nearest 10 cells. Each dot represents one cell and each color represents on method, with $\omega = \frac{\#\text{ inferred breakpoints}}{\#\text{ true breakpoints}}$ on the x-axis and breakpoint distance on the y-axis. Using multiple cells consistently outperforms individual analyses.

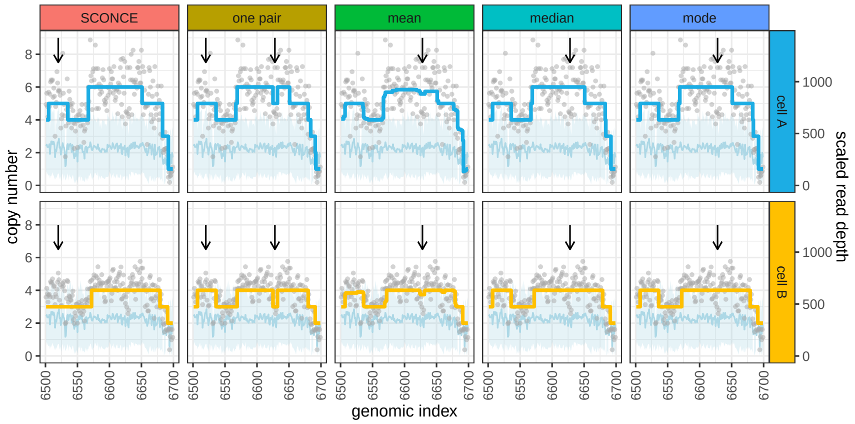

Figure S4: Improved CNA detection using multiple cells in real data [12]. Copy number calls and read depths are shown across methods for two cells (cell A: SRR054596, cell B: SRR054609, from [12]) for genomic windows 6500-6700 (x-axis). Genomic windows are 250kb across hg19 and numbered sequentially. Each dot shows scaled read depth (right y-axis, relative to diploid read depth) in one window, the light blue line and band show the mean and variance of the diploid read depth data, and colored lines show inferred copy number (left y-axis). Arrows denote differences in copy number calls between methods.

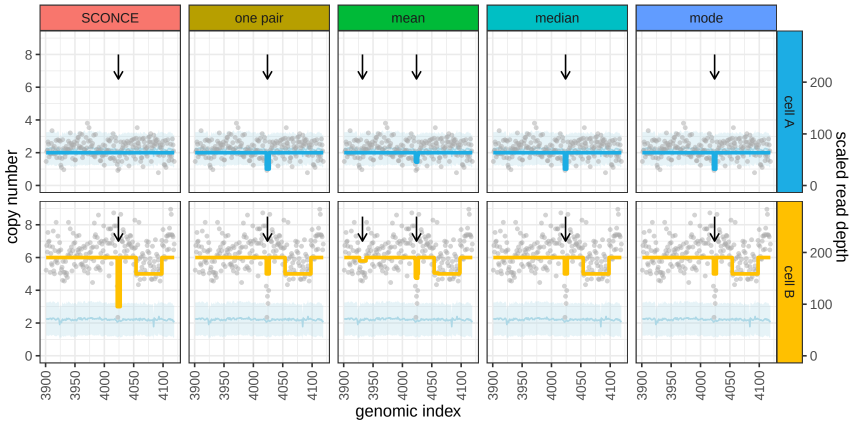

Figure S5: Improved CNA detection using multiple cells in real data [18]. Copy number calls and read depth are shown for two cells (cell A: cell 1538 with barcode TAGCCGGCAAGAACTA-1 from segment D, cell B: cell 1360 with barcode GCATACAAGTAACCCT-1. from segment B, from [18]) across methods in genomic windows 3900-4120 (x-axis). Windows are numbered sequentially across hg19 and are 250kb. Each dot shows per window read depth (right y-axis, scaled to the average diploid read depth), the light blue line and band show the mean and variance of the averaged diploid read depth data, and colored lines show inferred copy number (left y-axis). Arrows denote differences in copy number calls between methods.

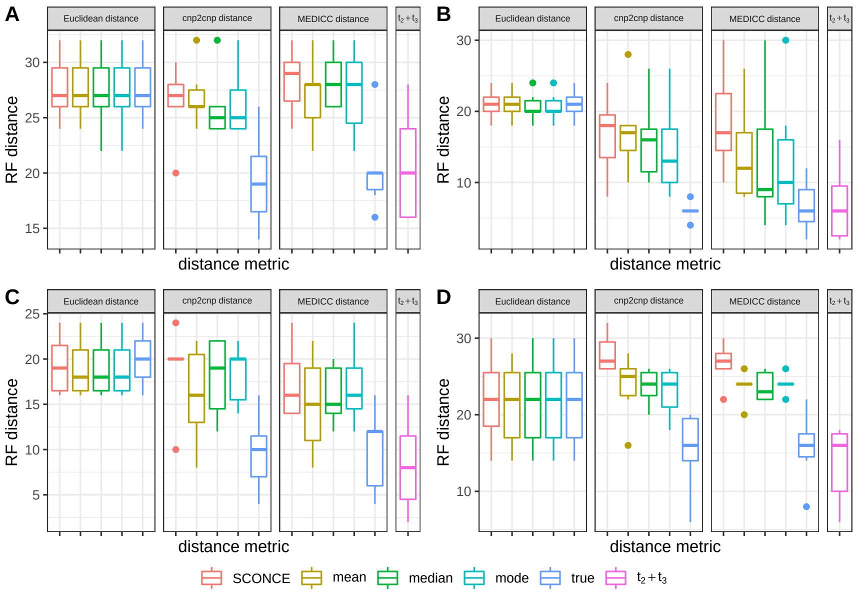

Figure S6: Robinson-Foulds (RF) distances for each simulation set (trees A-D) for different distance metrics. Distance metrics (Eucliden distance, cnp2cnp distance, MEDICC distance, $t_2 + t_3$) are grouped for each panel, and underlying CNPs sources are colored along the x-axis. Using $t_2 + t_3$ outperforms all other distance metrics on inferred copy number profiles.

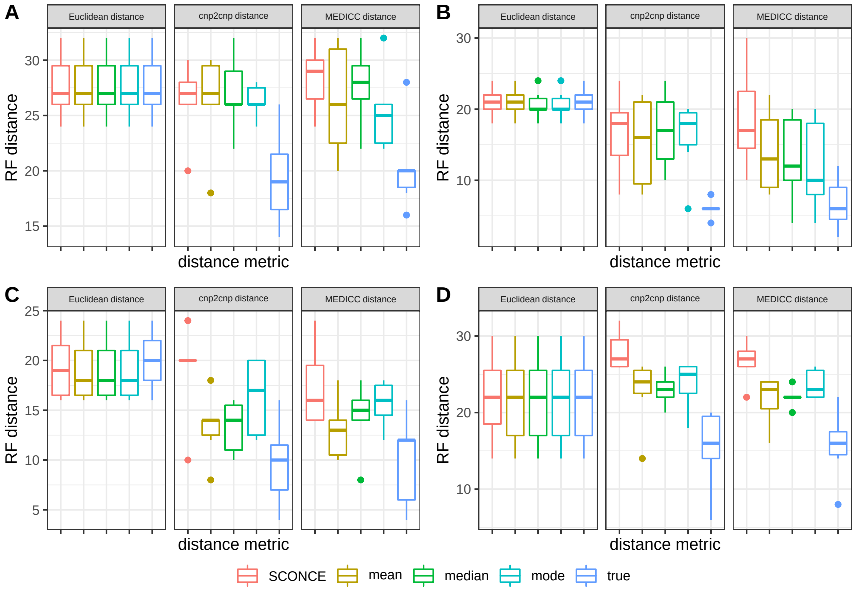

Figure S7: Robinson-Foulds distances for each simulation set (trees A-D) for different distance metrics, where consensus CNPs are summarized over each cell's 10 nearest neighbors. As in Supplementary Figure S6, distance metrics are grouped for each panel, and underlying CNPs types are colored along the x-axis. Note our $t_2 + t_3$ metric is excluded here; as a time saving measure, any pairs that aren't part of the nearest 10 of any cell (e.g., two very distant cells) are not analyzed, leading to missing data in the pairwise distance matrices.

Supplementary Tables

Simulation set/tree	Short description	Deletion rate, $\frac{\delta}{\tau_d}$	Insertion rate, $\frac{\varphi}{\tau_a}$	Mean deletion length, τ_d	Mean insertion length, τ_a
A	maximally imbalanced, ultrametric	0.025	0.025	10	10
B	perfectly balanced, ultrametric	0.025	0.025	10	10
C	maximally imbalanced, not ultrametric, all branches have equal length	0.05	0.065	10.5	8
D	maximally imbalanced, not ultrametric, terminal branch lengths decay logarithmically	0.05	0.065	10.5	8

Table S1: Description of simulation sets under the line segment model, relative to a genome length of 100. A range of tree structures were chosen to test both typical and edge cases.

	SCONCE	one pair	mean	median	mode	AneuFinder
A	1167	1006	394	1018.5	1019.5	1172
B	1009.5	796.5	91.5	959	959	1016.5
C	206	153	84	142	142	263
D	153.5	85	33	77	77.5	168.5

Table S2: Full median breakpoint distance values for all trees and methods.

	SCONCE	one pair	mean	median	mode	AneuFinder
A	0.4663	0.49	0.921	0.4862	0.4856	0.4623
B	0.4762	0.5	0.8643	0.4941	0.4897	0.4744
C	0.5134	0.5507	1.1667	0.5467	0.5436	0.4851
D	0.504	0.5349	1.0072	0.5346	0.5338	0.4885

Table S3: Full $\omega = \frac{\#\text{ inferred breakpoints}}{\#\text{ true breakpoints}}$ values for all trees and methods.

	nearest/most similar	10th nearest	furthest/least similar
A	88.0625	138.9585	205.853
B	87.0804	135.2585	208.149
C	26.9814	84.32675	127.957
D	38.76815	72.9452	112.81

Table S4: Median pairwise Euclidean distances between SCONCE profiles for each dataset. Median distances between the nearest/most similar cells, 10th nearest cells, and furthest/least similar cell are shown.

	SCONCE	one pair	mean	median	mode	AneuFinder
A	1167	1014	591	1009	1027	1172
B	1009.5	858	121	958.5	959.5	1016.5
C	206	152	90	130.5	135	263
D	153.5	84	38	70	76	168.5

Table S5: Full median breakpoint distance values for all trees and methods, where consensus methods summarize over the nearest 10 cells only.

	SCONCE	one pair	mean	median	mode	AneuFinder
A	0.4663	0.4907	0.8186	0.5155	0.4929	0.4623
B	0.4762	0.5	0.7833	0.5191	0.4941	0.4744
C	0.5134	0.5493	1.02	0.5786	0.5462	0.4851
D	0.504	0.5344	0.9027	0.5639	0.5304	0.4885

Table S6: Full $\omega = \frac{\#\text{ inferred breakpoints}}{\#\text{ true breakpoints}}$ values for all trees and methods, where consensus methods summarize over the nearest 10 cells only.

	SCONCE	mean	median	mode	true	$t_2 + t_3$
Euclidean distance	27	27	27	27	27	NA
cnp2cnp distance	27	26	25	25	19	NA
MEDICC distance	29	28	28	28	20	NA
$t_2 + t_3$	NA	NA	NA	NA	NA	20

(a) Tree A

	SCONCE	mean	median	mode	true	$t_2 + t_3$
Euclidean distance	21	21	20	20	21	NA
cnp2cnp distance	18	17	16	13	6	NA
MEDICC distance	17	12	9	10	6	NA
$t_2 + t_3$	NA	NA	NA	NA	NA	6

(b) Tree B

	SCONCE	mean	median	mode	true	$t_2 + t_3$
Euclidean distance	19	18	18	18	20	NA
cnp2cnp distance	20	16	19	20	10	NA
MEDICC distance	16	15	15	16	12	NA
$t_2 + t_3$	NA	NA	NA	NA	NA	8

(c) Tree C

	SCONCE	mean	median	mode	true	$t_2 + t_3$
Euclidean distance	22	22	22	22	22	NA
cnp2cnp distance	27	25	24	24	16	NA
MEDICC distance	27	24	23	24	16	NA
$t_2 + t_3$	NA	NA	NA	NA	NA	16

(d) Tree D

Table S7: Median Robinson-Foulds distances across different distance metrics on different CNPs for each simulation set.

	**SCONCE**	**mean**	**median**	**mode**	**true**
Euclidean distance	27	27	27	27	27
cnp2cnp distance	27	27	26	26	19
MEDICC distance	29	26	28	25	20

(a) Tree A

	**SCONCE**	**mean**	**median**	**mode**	**true**
Euclidean distance	21	21	20	20	21
cnp2cnp distance	18	16	17	18	6
MEDICC distance	17	13	12	10	6

(b) Tree B

	**SCONCE**	**mean**	**median**	**mode**	**true**
Euclidean distance	19	18	18	18	20
cnp2cnp distance	20	14	14	17	10
MEDICC distance	16	13	15	16	12

(c) Tree C

	**SCONCE**	**mean**	**median**	**mode**	**true**
Euclidean distance	22	22	22	22	22
cnp2cnp distance	27	24	23	25	16
MEDICC distance	27	23	22	23	16

(d) Tree D

Table S8: Median Robinson-Foulds distances across different distance metrics on different CNPs for each simulation set, where consensus CNPs are summarized over only the nearest 10 cells. Note that because summarizing over only the nearest 10 cells does not guarantee all pairs of cells will be jointly analyzed, no $t_2 + t_3$ values are given here, as estimating phylogenies using $t_2 + t_3$ requires a complete distance matrix.
